# Adequate magnesium level as an associated factor of pre-diabetes and diabetes mellitus remission in patients with obesity submitted to bariatric surgery

**DOI:** 10.1038/s41598-021-00584-0

**Published:** 2021-10-27

**Authors:** Vanessa Guerreiro, Isabel Maia, João Sérgio Neves, Daniela Salazar, Maria João Ferreira, Fernando Mendonça, Maria Manuel Silva, Sara Viana, Cláudia Costa, Jorge Pedro, Ana Varela, Eva Lau, Paula Freitas, Davide Carvalho

**Affiliations:** 1 Departamento de Endocrinologia, Diabetes e Metabolismo, Centro Hospitalar Universitário São João, Alameda Prof. Hernâni Monteiro, 4200-319 Porto, Portugal; 2 Faculdade de Medicina da Universidade do Porto, Porto, Portugal; 3 Instituto de Investigação e Inovação em Saúde, University of Porto, Porto, Portugal; 4 EPIUnit - Instituto de Saúde Pública, Universidade do Porto, Porto, Portugal; 5 Laboratório Para a Investigação Integrativa e Translacional em Saúde Populacional (ITR), Porto, Portugal; 6 Serviço de Medicina Interna, Unidade de Saúde Local do Norte Alentejo EPE, Alentejo, Portugal; 7 Serviço de Endocrinologia, Instituto Portugês de Oncologia, Porto, Portugal

**Keywords:** Diseases, Endocrinology

## Abstract

Bariatric surgery (BS) can lead to remission of type 2 diabetes *mellitus* (T2DM), however, the evidence on the influence of preoperative serum magnesium levels on this reversal is scarce. To study the influence of preoperative serum magnesium levels on the pre-T2DM and T2DM remission one year after BS. Retrospective study carried out among 1656 patients with obesity who underwent BS in the Centro Hospitalar Universitário São João. T2DM and pre-T2DM remission were defined as being normal glycaemic measures of at least one year’s after BS and without pharmacological therapy. To assess the association between preoperative serum magnesium levels and pre- and T2DM remission, logistic regression models, crude and adjusted for sex, age and body mass index were computed. Patients with normoglycaemia presented hypomagnesaemia less often than those patients with pre-T2DM and T2DM (17.0% vs. 21.3% vs. 39.9%) (*p* < 0.001). One year after BS, 62.9% of patients with pre-T2DM or T2DM before BS showed remission. Adequate magnesium levels were positively associated with T2DM and pre-T2DM remission, one year after BS (OR 1.79; 95% CI 1.34–2.38), independently of sex, age, and body mass index. Adequate preoperative serum magnesium levels showed to be an important clinical parameter for pre-T2DM and T2DM remission.

## Introduction

Obesity has become a global epidemic, whose consequences are well recognised and comprise several comorbidities, such as type 2 diabetes *mellitus* (T2DM)^1^. Treatment for obesity includes lifestyle modifications, pharmacological therapy, and bariatric surgery (BS), the latter being the most effective treatment^2^.

T2DM is associated with micronutrients metabolism alteration. Specifically, patients with T2DM commonly present magnesium deficiency, at a prevalence of approximately 30%^3^. This may result, for example, from poor oral intake, poor gastrointestinal absorption, and enhanced renal magnesium excretion. Furthermore, hypomagnesaemia, might also influence insulin secretion and contribute to insulin resistance, thus creating a vicious circle^4^.

Magnesium is the second most abundant intracellular element, having a widespread role in the body^5^. It is an essential cofactor of several enzymes involved in the carbohydrate, lipid and protein metabolits, and is also important for the response to certain hormones, such as parathormon^6^.

It is well known that, when compared with conventional medical therapy, BS greatly decreases the prevalence of obesity-related comorbidities, and in particular it can lead to the improvement and remission of T2DM in individuals with obesity^2^, however, data on the impact of magnesium levels on this reversal is scarce. A previous study reported an increase in serum magnesium levels only in patients in which diabetes resolved after surgery^7^. Another study demonstrated that patients without T2DM remission after BS started from an inferior level of magnesium, being those patients were submitted only to Roux-en-Y gastric bypass (RYGB)^8^. Even that, data regarding the relationship between preoperative serum magnesium levels and T2DM remission after different types of BS are limited.

Therefore, this study aimed to assess the relationship between the glycaemic profile of patients with obesity submitted to BS and preoperative magnesium serum levels and to study its influence on pre-T2DM and T2DM remission one year after BS.

## Materials and methods

### Study design and participants

We carried out a retrospective cohort study including 2520 morbidly patients with obesity submitted to BS – RYGB, laparoscopic adjustable gastric band (LAGB), or laparoscopic sleeve gastrectomy (LSG)—at the Centro Hospitalar Universitário São João, Porto, between January 2010 and July 2018. Data were obtained from patients’ clinical file. All study participants either had body mass index (BMI) > 40 kg/m^2^, or obesity-related comorbidities and BMI > 35 kg/m^2^, and all had complied with a dietary plan for at least 12 months. Patients with data from preoperative and at one-year post-surgery clinical visits were considered. Patients without data on preoperative serum magnesium levels or glycaemic profile were excluded, resulting in a final sample of 1656 patients.

All procedures performed in the study were approved by the Ethics Committee for Health of Centro Hospitalar Universitário de São João. For this type of study formal consent is not required in accordance with the national legislation and the institutional requirements.

### Socio-demographic and clinical parameters

Data on age, sex, weight, height, waist (WC) and hip circumferences (HC), blood pressure (BP), the type of BS performed (LAGB, RYGB or LSG), and on magnesium supplementation were collected. BMI was calculated as weight (kg) divided by height (m) squared.

Data on plasma glucose, glycated haemoglobin (A1c), and insulin in serum were also collected. Preoperative serum magnesium levels were measured by spectrophotometry with xylidyl blue. HOMA-IR [(fasting serum glucose × fasting serum insulin)/405] was used as a measure of insulin resistance^9^, and HOMA-β [(20 × fasting serum insulin)/[(fasting serum glucose) – 3.5)]] as a measure of β-cell function^9^.

The American Diabetes Association guidelines^10^ criteria were used to define T2DM, namely: A1c ≥ 6.5% (7.8 mmol/L), fasting plasma glucose ≥ 126 mg/dL or 2 h post-load plasmatic glucose ≥ 200 mg/dL during an oral glucose tolerance test; and for pre-T2DM: 6.5 mmol/L (5.7%) ≤ A1c ≤ 6.4% (7.6 mmol/L) or 100 mg/dL > fasting glucose < 126 mg/dL. Patients using antidiabetic treatment were considered as having T2DM. Preoperative serum magnesium levels below or equal to 1.5 mEq/L is defined as hypomagnesaemia, while above 1.5 mEq/L is considered as adequate levels^11^.

Complete diabetes remission was considered if the patient had pre-T2DM or T2DM before BS and normal glycaemic measures (A1C < 5.7%—6.5 mmol/L and fasting plasma glucose < 100 mg/dL), as well as no active pharmacologic therapy one year after BS^12^.

### Statistical analysis

Continuous variables were described as the mean and standard deviation (SD), or as the median and percentiles 25 and 75 (P25; P75), as appropriate. For normally-distributed continuous variables, ANOVA was used, while for non-normally distributed continuous variables, the Mann–Whitney U test and Kruskal–Wallis test were used. Categorical variables were described as absolute and relative frequencies and were compared using the Chi-square test.

Logistic regression models were performed to evaluate the association between preoperative serum magnesium levels (hypomagnesaemia vs. adequate) and pre- and T2DM remission one year after BS. Sensitivity analyses were also performed, namely on assessing the association between preoperative serum magnesium levels and the occurrence of T2DM and another analysis on exploring the association between preoperative serum magnesium levels (hypomagnesaemia vs. adequate) and only T2DM remission one year after BS. For all of the analyses, crude and adjusted odds ratios (ORs) and respective 95% confidence intervals (95%CIs) were computed. The final models included as independent variables sex, age and BMI, based on previously described confounding variables and/or on statistical significant crude associations.

Statistical analysis was carried out using SPSS Statistics 25.0 (IBM Corp, Armonk, New York). A significance level of 5% was considered.

### Ethical approval

This study was approved by the Ethics Committee for Health our centre, Centro Hospitalar Universitário de São João.

### Consent to participate

For this type of study formal consent is not required in accordance with the national legislation and the institutional requirements.

### Consent do publish

For this type of study formal consent is not required in accordance with the national legislation and the institutional requirements.

## Results

Demographic and preoperative characteristics of the studied population are presented in Table 1. The studied sample had a mean age of 42.5 ± 10.7 years, and the majority of patients were women (85.8%). With regards to BS, 56.9% of the participants were submitted to RYGB, 28.6% to LSG, and 14.5% to LAGB. The mean of preoperative BMI was 43.8 ± 5.7 kg/m^2^, WC 123.0 ± 13.2 cm, and HC 132.3 ± 11.6 cm (Table 1).

**Table 1:** Preoperative characteristics of the studied sample.

n (%)	N	Total population	Normoglycaemic	Pre-T2DM	T2DM	*p* value
		1656 (100.0)	(588 (35.5))	582 (35.1)	486 (29.3)	
Age (years) [mean (SD)]	1655 (99.9)	42.5 (10.7)	37.7 (9.7)	43.0 (10.1)	47.8 (9.9)	< 0.001
Gender (n (%))	1655 (99.9)					0.005
Men		235 (14.2)	70 (11.9)	75 (12.9)	90 (18.5)	
Women		1420 (85.8)	517 (88.1)	507 (87.1)	396 (81.5)	
BMI [kg/m2; mean (SD)]	1647 (99.5)	43.8 (5.7)	43.1 (5.3)	44.0 (5.4)	44.5 (6.3)	< 0.001
Waist circumference [cm; mean (SD)]	1304 (78.7)	123.0 (13.2)	119.8 (12.3)	123.5 (12.9)	126.2 (13.7)	< 0.001
Hip circumference [cm; mean (SD)]	1232 (74.4)	132.3 (11.6)	132.4 (10.4)	132.6 (11.1)	131.8 (13.6)	0.650
SBP [mmHg; mean (SD)]	1343 (81.1)	135.1 (17.3)	130.8 (16.4)	136.8 (17.4)	138.1 (17.4)	< 0.001
DBP [mmHg; mean (SD)]	1344 (81.2)	83.6 (11.1)	82.2 (10.8)	84.1 (11.1)	84.4 (11.4)	0.006
Glucose [mg/dL; median (P25;P75)]	1564 (94.4)	93.5 (84.0;106.8))	86.0 (79.0;92.0))	96.0 (87.0;103.0)	115.0 (97.0;147.0)	< 0.001
Glucose 120' [mg/dL; mean (SD)]	1144 (69.1)	136.1 (45.0)	108.1 (19.6)	137.5 (29.4)	195.7 (57.8)	< 0.001
A1c [%; median (P25;P75) ]	1597 (96.4)	5.6 (5.3;6.0))	5.3 (5.1;5.5))	5.7 (5.5;5.9)	6.4 (5.8;7.1)	< 0.001
HOMA-IR [median (P25;P75)]	1355 (81.8)	4.0 (2.7;6.4))	3.2 (2.2;4.6)	4.2 (2.8;6.2)	6.3 (3.5;9.6)	< 0.001
HOMA-β [median (P25;P75)]	1355 (81.8)	212.8 (125.4;348.2)	252.8 (160.7;402.2)	214.7 (127.9;348.7))	153.0 (87.9;264.2)	< 0.001
Insulin µU/mL; median (P25;P75)]	1441 (87.0)	17.8 (11.9;26.2)	15.5 (11.0;22.4)	18.6 (12.2;26.5)	20.5 (13.7;31.2)	< 0.001
Magnesium level [n (%)]	1656 (100.0)					< 0.001
Hypomagnesemia (≤ 1.5)		418 (25.2)	100 (17.0)	124 (21.3)	194 (39.9)	
Normomagnesemia (> 1.5)		1,238 (74.8)	488 (83.0)	458 (78.7)	292 (60.1)	
Type of bariatric surgery [n (%)]	1656 (100.0)					0.004
LAGB		240 (14.5)	59 (10.0)	97 (16.7)	84 (17.3)	
RYGB		943 (56.9)	347 (59.0)	323 (55.5)	273 (56.2)	
LSG		473 (28.6)	182 (31.0)	162 (27.8)	129 (26.5)	
Magnesium supplementation	1617 (97.6)					0.427
No		1586 (98.1)	564 (98.6)	555 (98.1)	467 (97.5)	
Yes		31 (1.9)	8 (1.4)	11 (1.9)	12 (2.5)	

A1c: glycated haemoglobin; BMI: body mass index; DBP: diastolic blood pressure; IQR, interquartile range; HOMA-IR: homeostasis model assessment of insulin resistance; HOMA-β: homeostasis model assessment of β-cell function; LAGB: laparoscopic gastric band; LSG: sleeve gastrectomy; P25, percentile 25; P75; percentile 75; RYGB: Roux-en-Y gastric bypass, SBP, systolic blood pressure; SD, standard deviation, T2DM, Type 2 Diabetes Melitus.

Before BS, a lower proportion of women had T2DM (*p* = 0.005). Additionally, compared to normoglycaemic, patients with T2DM were older (47.8 (9.9) years; *p* < 0.001) and presented a higher mean BMI of 44.5 ± 6.3 kg/m^2^ (*p* < 0.001).

The preoperative serum magnesium levels varied according to the glycaemic profile. Hypomagnesaemia was present in 39.9% of the patients with T2DM and among 21.3% and 17.0% of the patients with pre-T2DM and with normoglycaemia, respectively (*p* < 0.001) (Table 1).

Higher glucose levels was observed in patients with hypomagnesaemia across categories of the glycaemic profile, particularly in patients with pre-T2DM and T2DM. Also, in general, higher HbA1c, HOMA-IR and lower HOMA-β was verified in those patients with hypomagnesaemia, however, only statistically significant for the total and T2DM patients (Table 2).

**Table 2:** Correlations between magnesium levels and glycaemic indicators according to the glycaemic profile.

	Total population			Normoglycaemic			Pre-T2DM			T2DM		
	Hypo-magnesemia (≤ 1.5)	Normo-magnesemia (> 1.5)	*p*	Hypo-magnesemia (≤ 1.5)	Normo-magnesemia (> 1.5)	*p*	Hypo-magnesemia (≤ 1.5)	Normo-magnesemia (> 1.5)	*p*	Hypo-magnesemia (≤ 1.5)	Normo-magnesemia (> 1.5)	*p*
Glucose (mg/dL)*	101.0 (88.0;134.0)	92.0 (83.0;101.0)	< 0.001	86.0 (81.0;93.0)	86.0 (79.0;91.75)	0.088	100.0 (89.5;105.5)	95.0 (87.0;102.0)	0.014	135.5 (105.2;173.0)	109.0 (94.0;131.0)	< 0.001
HbA1c (%)*	5.8 (5.4;6.7)	5.6 (5.3;5.9)	< 0.001	5.3 (5.0;5.5)	5.3 (5.1;5.5)	0.502	5.7 (5.4;5.9)	5.7 (5.5;5.9)	0.066	6.8 (6.1;8.1)	6.1 (5.7;6.6)	< 0.001
HOMA-IR*	4.8 (2.9;7.6)	3.9 (2.6;6.1)	0.007	3.3 (2.2;4.9)	3.1 (2.1;4.5)	0.427	4.3 (2.9;6.2)	4.1 (2.8;6.3)	0.475	7.2 (4.2;11.5)	5.8 (3.4;8.7)	0.007
HOMA-β*	186.3 (105.1;309.3)	221.3 (129.8;362.1)	0.033	252.8 (153.2;384.4)	252.8 (163.8;403.1)	0.501	202.5 (134.2;319.3)	218.1 (127.4;358.1)	0.342	120.4 (69.2;249.7)	160.4 (96.0;276.6)	0.033

*Median (P25, P75).

It was observed that adequate magnesium levels was negatively associated with having T2DM (OR = 0.34; 95% CI: 0.26–0.43), independently of sex, age and BMI (Table 3).

**Table 3:** Association between serum magnesium levels and T2DM.

n (%)	T2DM		Crude OR (95%CI)	Adjusted OR (95%CI)*
	No	Yes		
	1170 (70.7)	486 (29.3)		
**Preoperative magnesium levels**				
Hypomagnesemia (≤ 1.5)	224 (19.1)	194 (39.9)	1	1
Normomagnesemia (> 1.5)	946 (80.9)	292 (60.1)	0.36 (0.28–0.45)	0.34 (0.26–0.43)

* Adjusted for sex, age and body mass index.

One year after BS, 62.9% of patients with pre-T2DM or T2DM, before BS, showed remission. After adjustments, compared with patients with hypomagnesaemia, those with adequate magnesium levels were almost two times more likely to have remission of T2DM and pre-T2DM one year after the BS (OR = 1.79; 95% CI: 1.34–2.38). Similar results were obtained, when only T2DM remission was considered (OR = 1.93; 95% CI:1.29–2.88) (Table 4).

**Table 4:** Association between serum magnesium levels and pre-T2DM and T2DM remission, and only T2DM remission.

n (%)	Remission		Crude OR (95%CI)	Adjusted OR (95%CI)*
	No	Yes		
	396 (37.1)	672 (62.9)		
*Pre-T2DM and T2DM*				
**Preoperative magnesium levels**				
Hypomagnesemia (≤ 1.5)	147 (37.1)	171 (25.4)	1	1
Normomagnesemia (> 1.5)	249 (62.9)	501 (74.6)	1.73 (1.32–2.26)	1.79 (1.34–2.38)
*Only T2DM* (n = 486)				
**Preoperative magnesium levels**				
Hypomagnesemia (≤ 1.5)	128 (46.2)	66 (31.6)	1	1
Normomagnesemia (> 1.5)	149 (53.8)	143 (68.4)	1.86 (1.28–2.71)	1.93 (1.29–2.88)

CI, confidence interval; OR, odds ratio; T2DM, type 2 diabetes* mellitus*.

*Adjusted for sex, age and body mass index.

## Discussion

Among the sample of patients with obesity under study, we verified that hypomagnesaemia were frequent in patients with T2DM, and that preoperative serum magnesium levels were related to glucose parameters. According to our findings, patients with adequate preoperative serum magnesium levels had a higher odds of T2DM and pre-T2DM remission. The inverse association between levels of magnesium and T2DM remission has also been previously reported in one study, however, it only included patients submitted to RYGB^8^.

Our results are in accordance with previous studies^13,14^, which pointed out that hypomagnesaemia or lower levels of magnesium occurred frequently in patients with T2DM. Although this reduced levels of magnesium among T2DM could occur due to differences between the patients studied, such as metabolic differences^13^, it is well established that circulating magnesium levels tend to decrease in patients with T2DM, especially in those who are poorly controlled^7,15^.

We observed that, in general, preoperative serum magnesium levels are related with glucose parameters according to glycaemic profile of the patients. These findings were in accordance with previous data on the association between magnesium status and different glucometabolic variables, whereby the serum magnesium concentrations have shown to be inversely related to circulating glucose concentrations and to insulin resistance—both in patients with diabetes (BMI of 26.7 ± 0.5 kg/m^2^) and in those without diabetes (BMI of 26.9 ± 0.4 kg/m^2^)^16,17^. In the literature, it has been reported that hypomagnesaemia can affect both insulin secretion and insulin action, although the mechanisms through which hypomagnesaemia can induce or worsen existing T2DM are not fully understood^18^. Furthermore, insulin can increase renal magnesium excretion. Also, hyperglycaemia increases renal magnesium and glucose excretion, which could contribute to hypomagnesaemia and therefore there is a bi-directional relationship between both pathologies, which creates a vicious circle that worsens the glucometabolic status^4,19^. Despite the role of magnesium in the regulation of electrical activity and insulin secretion by pancreatic β-cells^20^, those studies which evaluated the relationship between magnesium levels and insulin secretion led to diverging conclusions, with some of them demonstrating that hypomagnesaemia is associated with hyperinsulinemia^17,21^ and others showing impairment in insulin secretion^22,23^. However, it was demonstrated that in hypomagnesaemia, intracellular levels of magnesium, adenosine triphosphate (ATP), and especially of Mg^2+^-ATP complex (MgATP) decrease, which lead to the inadequate activation of the sulfonylurea receptor 1 (SUR1) channel subunit and, ultimately, to the increase in the basal secretion of insulin^20^. These discrepancies could be due to the different adaptability of the β-cell in the studied populations.

According to our findings, almost two-thirds of the patients with pre- or T2DM before BS showed remission one year after surgery, being observed similar results when considering only patients with T2DM remission. Adequate preoperative magnesium levels revealed to be positively associated with that remission, regardless of sex, age and BMI of the patients. According to the literature, T2DM remission could occur in as many as 80% of cases, but not in all of the patients submitted to BS^24^. The metabolic effects of BS appear to be partly independent of weight loss, occurring earlier and without a direct relation with this magnitude^25^. However, the underlying mechanisms for why this occurs remain unclear and multiple pathways have been proposed, e.g., due to hormonal changes that occur as a result of BS^26^. Age, preoperative A1C, use of insulin, and the type of oral anti-diabetic medication could predict diabetes remission after BS^27^, but other factors that may influence this remission have been identified^28^.

The findings of this study are in line with previous data that analysed factors associated with T2DM remission after BS^8^. However, despite the evidence of a close relationship between magnesium levels and metabolic control in T2DM, these previous studies did not explore the influence of preoperative serum magnesium levels on T2DM remission.

There are limitations in our study which need to be mentioned. Although there was a relationship between preoperative magnesium levels and the remission of diabetes after BS, the causal mechanisms still need to be explored, since magnesium intake levels, renal excretion and intracellular concentrations were not evaluated in this study. No data on the erythrocyte magnesium content was available in our study. Magnesium is a predominantly intracellular ion, and, therefore, its serum concentration may not accurately reflect magnesium status or the intracellular pool, however, it was found that serum magnesium exhibits a good correlation with intracellular magnesium^29^ and this potential limitation presumably did not influence our conclusions. Furthermore, it is not known whether hypomagnesaemia is a cause or consequence of worse metabolic control in T2DM^19^, which could influence our results, as patients with worse metabolic control have less DM remission (30). The patients without T2DM remission could have low levels of magnesium due to longer diabetes duration and a more deteriorated glucometabolic status, which can contribute do lower T2DM remission (30), however, we know that magnesium has an important role in the insulin action (18) and although these factors may have influenced them, they will certainly not be the only explanation of the observed data. Nonetheless, this study was strengthened by a large number of subjects included, who were consecutively selected at one hospital, and also because the sample included patients who had undergone different surgical techniques (malabsorptive and restrictive procedures). Furthermore, considering the paucity of research on the influence of preoperative serum magnesium levels in pre-T2DM and T2DM remission after different types of BS, this study provided relevant insights on this regard.

Nevertheless, further longitudinal studies are needed to clarify underlying mechanisms between the association between magnesium concentrations and T2DM remission.

## Conclusion

Patients with morbid obesity and T2DM or pre-T2DM had frequently hypomagnesemia and an inverse association between glucose parameters and magnesium status was observed.

One year after BS 62.9% of obese patients showed pre-T2DM or T2DM remission. Adequate preoperative serum magnesium levels showed to be an important clinical parameter for pre-T2DM and T2DM remission.

## Data Availability

The datasets generated during and/or analyzed during the current study are available from the corresponding author on reasonable request.
